# A Sirtuin‐1‐Targeted Gene‐Activating Tetrahedral DNA Attenuates Bladder Fibrosis by Restoring Mitophagy in Fibroblasts via the SIRT1‐FOXO3‐BNIP3 Axis

**DOI:** 10.1002/advs.202519527

**Published:** 2025-11-23

**Authors:** Wei Wang, Ran Yan, Lede Lin, Lei Xiang, Xiaozhi Xia, Liao Peng, Xiaoshuai Gao, Jiawei Chen, Yang Xiong, Liang Zhou, Yunfeng Lin

**Affiliations:** ^1^ Department of Urology Institute of Urology (Laboratory of Reconstructive Urology) West China Hospital Sichuan University Chengdu Sichuan 610041 P. R. China; ^2^ State Key Laboratory of Oral Diseases National Clinical Research Center for Oral Diseases West China Hospital of Stomatology Sichuan University Chengdu 610041 P. R. China

**Keywords:** bladder fibrosis, fibroblast, sirtuin‐1, small‐activating RNA, tetrahedral framework nucleic acid

## Abstract

Bladder fibrosis represents a widespread global health challenge associated with substantial socioeconomic burden. To date, no effective therapeutic interventions are available to halt or reverse its progression. Small activating RNA (saRNA)‐based therapy has recently garnered increasing interest due to its high target specificity and potent efficacy. Nevertheless, the clinical translation of saRNA is hampered by inherent limitations including structural instability, nuclease sensitivity, and inefficient cellular internalization. In this study, single‐cell and bulk transcriptomic analyses are integrated, which reveal that SIRT1 is the only sirtuin family member significantly downregulated in both fibrotic bladder tissues and activated fibroblasts. To address this, a tetrahedral DNA functionalized with saRNA targeting SIRT1 activation is engineered, termed TSA. TSA exhibits exceptional biocompatibility and markedly attenuates bladder dysfunction and fibrotic remodeling in a bladder outlet obstruction model. Mechanistically, TSA administration robustly restores SIRT1 expression, facilitating FOXO3A deacetylation and alleviating its transcriptional repression of BNIP3. This cascade leads to the activation of PINK1‐PARKIN‐mediated mitophagy, suppresses mitochondrial reactive oxygen species accumulation, and ultimately leads to the inhibition of fibroblast activation and collagen deposition. These compelling findings underscore the therapeutic potential of TSA as a promising strategy for the treatment of bladder fibrosis, with broad implications for clinical application.

## Graphical abstract

The SIRT1‐targeted saRNA‐delivering tetrahedral DNA (TSA) treatment effectively upregulates SIRT1 expression, which subsequently promotes FOXO3A deacetylation. This deacetylation event relieves FOXO3A's transcriptional repression on the BNIP3 gene, thereby initiating PINK1‐PARKIN‐dependent mitophagy. The enhancement of mitophagic flux ultimately reduces mitochondrial reactive oxygen species accumulation, resulting in the inhibition of fibroblast activation and pathological collagen deposition.

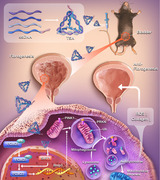

## 1 Introduction

Fibrosis represents a pathological process characterized by tissue repair and scarring, strongly associated with chronic inflammatory responses and other underlying mechanisms.^[^ ^1^ ^]^ Within the field of urology, bladder dysfunction is frequently linked to fibrotic remodeling.^[^ ^2^ ^]^ The development of fibrosis in the bladder leads to impaired contractility of the detrusor muscle, resulting in the inability to voluntarily void among affected patients. These structural alterations are most commonly observed in cases of bladder outlet obstruction (BOO).^[^ ^3^ ^]^ Global prevalence estimates indicate that BOO affects ≈21.5% of adults aged 20 years and above as of 2008, with a projected increase to 21.8% by 20 18.^[^ ^4^ ^]^ A significant number of patients continue to experience lower urinary tract symptoms even after surgical relief of the obstruction, suggesting the establishment of irreversible pathological remodeling in bladder architecture.^[^ ^5^ ^]^ Currently, no effective therapeutic interventions are available to halt or reverse BOO‐induced tissue remodeling. Consequently, there is a critical need to explore novel pharmacological agents capable of mitigating bladder dysfunction associated with maladaptive tissue remodeling.

The sirtuin family (SIRT1‐7) represents a class of evolutionarily conserved NAD+‐dependent deacetylases, which play pivotal roles in epigenetic regulation and metabolic homeostasis.^[^ ^6^ ^]^ These enzymes share a conserved catalytic core that requires nicotine adenine dinucleotide (NAD+) as an essential cofactor, yet they exhibit distinct substrate specificities and subcellular localizations, enabling their participation in diverse biological processes. Accumulating evidence has implicated aberrant sirtuin signaling in the pathogenesis of multiple human diseases, including tissue fibrosis, aging‐related degeneration, metabolic syndrome, carcinogenesis, and neurodegenerative disorders.^[^ ^6^ ^]^ For instance, emerging evidence indicates that SIRT1 activation exacerbates profibrotic responses to transforming growth factor‐β1 (TGF‐β1), as reflected by augmented Smad reporter activity, upregulation of TGF‐β1‐dependent gene transcription, and increased collagen secretion.^[^ ^7^ ^]^ Furthermore, Zhang et al. demonstrates that SIRT3 modulates mitochondrial protein acetylation and metabolic homeostasis in tubular epithelial cells during renal fibrosis.^[^ ^8^ ^]^ Another research has also highlighted the critical involvement of SIRT6 in hepatic stellate cell activation and liver fibrogenesis through deacetylation of the pro‐fibrotic transcription factor Smad2.^[^ ^9^ ^]^ Although members of the SIRT family are increasingly recognized as key regulators of fibrotic pathogenesis across various organ systems, their precise roles and mechanisms in bladder fibrosis remain inadequately characterized.

RNA therapeutics represent an emerging class of gene‐modulating agents, which encompass a range of nucleic acid‐based drugs including small interfering RNA (siRNA) and microRNA (miRNA) designed for targeted gene suppression.^[^ ^10^ ^]^ A significant limitation of siRNA and miRNA approaches, however, lies in their inability to enhance gene expression.^[^ ^10^ ^]^ To address this constraint, small activating RNAs (saRNAs) have been developed as a promising alternative.^[^ ^11^ ^]^ Structurally, saRNAs are double‐stranded RNA molecules typically composed of ≈20 nucleotides. Mechanistically, whereas siRNAs facilitate the formation of the RNA‐induced silencing complex (RISC) to degrade complementary mRNA and suppress gene expression, saRNAs assemble into an RNA‐induced transcriptional activation (RITA) complex.^[^ ^12^, ^13^ ^]^ This complex promotes sustained transcriptional upregulation of target genes, thereby bridging a critical gap in RNA‐based strategies for gene activation.^[^ ^12^ ^]^ Despite their considerable potential in transcriptional enhancement, the broad application of saRNAs remains constrained by several inherent limitations. These include inadequate stability in serum, high susceptibility to enzymatic degradation by RNases, and poor permeability across cellular membranes, all of which currently impede their efficacy as reliable gene‐regulating therapeutics.^[^ ^14^, ^15^ ^]^

Tetrahedral framework nucleic acids (tFNAs) serve as an efficient and versatile delivery platform for small activating RNA (saRNA).^[^ ^16^, ^17^, ^18^, ^19^ ^]^ These systems are synthesized through the self‐assembly of four specifically designed DNA oligonucleotides, guided by Watson–Crick base pairing principles, into a stable 3D configuration.^[^ ^20^, ^21^ ^]^ In contrast to conventional lipid‐based vectors such as liposomes, tFNAs demonstrate markedly reduced cytotoxicity and superior biocompatibility.^[^ ^22^, ^23^ ^]^ Beyond their role as nucleic acid carriers, tFNAs possess inherent bioactivities, including scavenging of reactive oxygen species (ROS) and modulation of inflammatory responses.^[^ ^24^, ^25^ ^]^ In the present study, integrated analysis of single‐cell and bulk transcriptomic data revealed that SIRT1 was uniquely downregulated among sirtuin family members in fibrotic bladder specimens and activated fibroblasts. To address this deficiency, we engineered a tetrahedral nucleic acid‐based systems, designated TSA, functionalized with saRNA targeting SIRT1 for transcriptional activation. Both in vitro and in vivo assessments confirmed that TSA administration effectively upregulated SIRT1 expression, induced FOXO3A deacetylation, and consequently attenuated its transcriptional repression of BNIP3. This cascade activated the PINK1‐PARKIN‐mediated mitophagy pathway, suppressed mitochondrial reactive oxygen species (mtROS) accumulation, and ultimately led to the inhibition of fibroblast activation and collagen deposition. Our findings establish a novel strategy to enhance the delivery efficiency and therapeutic efficacy of disease‐modifying biological agents for bladder fibrosis, offering substantial potential for clinical translation and future applications in the management of fibrotic uropathies.

## 2 Results and Discussion

### 2.1 SIRT1 is Downregulated in Fibrotic Bladder Tissue and Activated Fibroblasts

The SIRT family represents a class of evolutionarily conserved NAD⁺‐dependent deacylases, widely present across eukaryotic, archaeal, and eubacterial species, with particularly well‐characterized roles in mammalian systems.^[^ ^26^ ^]^ These enzymes function as crucial metabolic sensors and multifunctional regulators, orchestrating a diverse array of cellular processes such as energy homeostasis, DNA damage repair, mitochondrial bioenergetics and dynamics, telomere integrity, inflammatory signaling, redox balance, and programmed cell death.^[^ ^26^ ^]^ In mammals, the SIRT family is categorized into seven distinct members (SIRT1 through SIRT7), each exhibiting unique subcellular localization, catalytic specificity, and biological functions.^[^ ^6^ ^]^ Dysregulation of SIRT expression and activity has been strongly implicated in the pathophysiological mechanisms underlying a spectrum of diseases, including fibrotic conditions, age‐associated degeneration, obesity, diabetes mellitus, carcinogenesis, and neurodegenerative disorders.^[^ ^6^ ^]^ Despite the established regulatory roles of the SIRT family in fibrotic processes across multiple organs, their specific functions in bladder fibrosis remain poorly elucidated.

**Figure 1:**
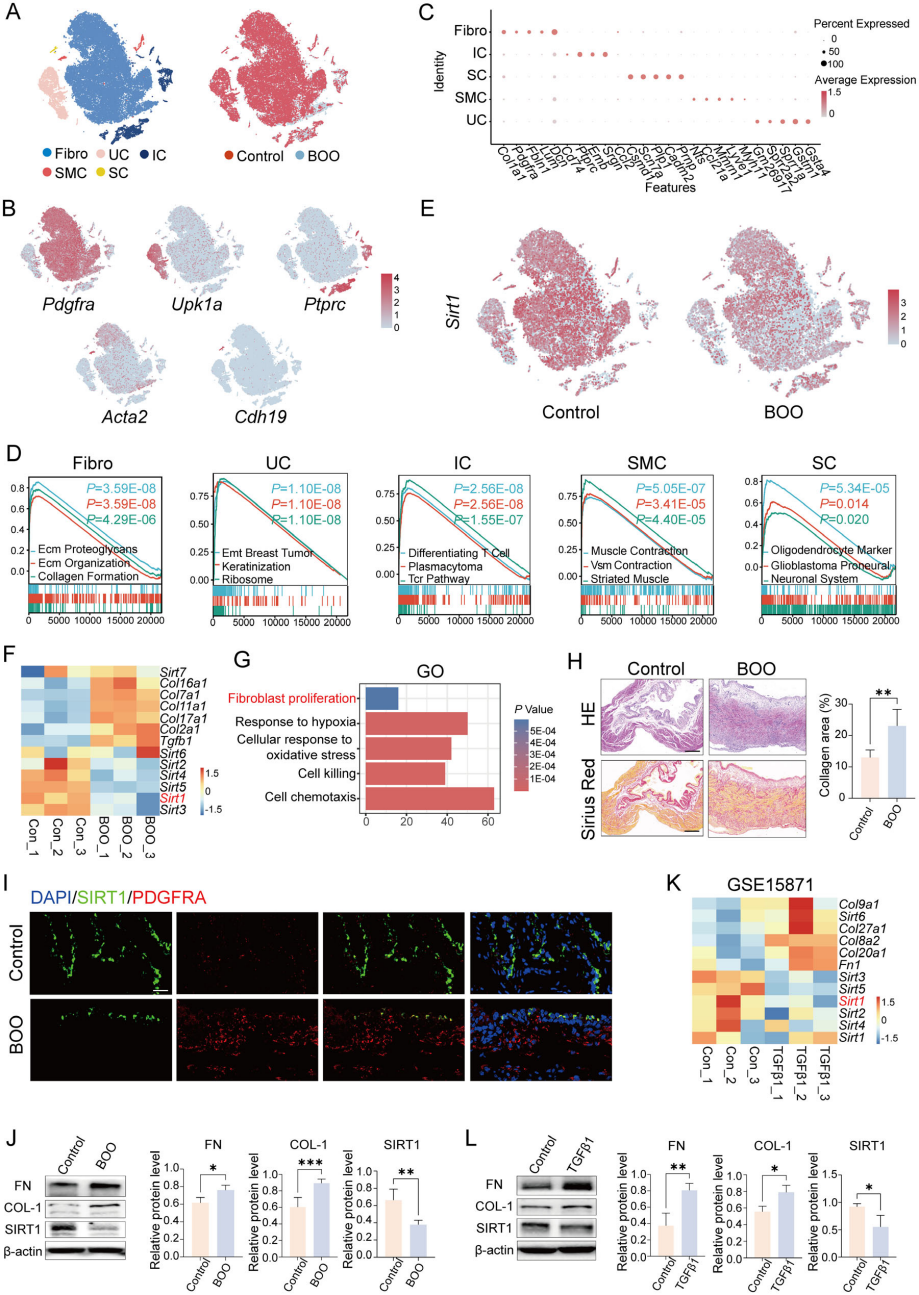
SIRT1 is downregulated in Fibrotic Bladder Tissue and Activated Fibroblasts. A) t‐distributed stochastic neighbor embedding (t‐SNE) visualization illustrating the distribution of five distinct cell populations isolated from murine bladder tissues in both control and BOO‐induced groups (*n* = 3 per group). Fibro, fibroblast; UC, urothelial cell; IC, immune cell; SMC, smooth muscle cell; SC, Schwann cell. B) Gene expression patterns of marker genes on t‐SNE plots: fibroblasts (*Pdgfra*), urothelial cells (*Upk1a*), immune cells (*Ptprc*), smooth muscle cells (*Acta2*), and Schwann cells (*Cdh19*). C) Dot plot summarizing the top five marker genes in each cluster. D) Gene Set Enrichment Analysis (GSEA) highlighting biological processes significantly associated with marker genes derived from each cell population. E) Feature plot analysis reveals pronounced downregulation of *Sirt1*expression in fibrotic bladder tissue. F,G) Bulk RNA sequencing analysis of a rat BOO model identifies differentially expressed genes (DEGs) enriched in pathways related to fibroblast proliferation, concurrent with reduced *Sirt1*expression. H) Representative histopathological images of bladder sections stained with hematoxylin and eosin (H&E) and Sirius red, comparing control and BOO groups (scale bar = 100 µm). Data are presented as the mean ± standard deviation (SD) (*n* = 3). I) Immunofluorescence staining confirms predominant SIRT1 expression within PDGFRA⁺ fibroblasts and demonstrates significant reduction following BOO induction (scale bar = 20 µm). J) Western blot analysis shows decreased SIRT1 protein levels accompanied by elevated expression of collagen‐I (COL‐1) and fibronectin (FN) in fibrotic bladders. K) Integrated analysis of the publicly available transcriptomic dataset GSE15871 (Gene Expression Omnibus) reveals significant downregulation of *Sirt1*in TGF‐β1‐stimulated fibroblasts relative to controls. Data are presented as the mean ± SD (*n* = 3). L) Validation of SIRT1 suppression in cultured mouse bladder fibroblasts following TGF‐β1 treatment. Data are presented as the mean ± SD (*n* = 3). Statistical analysis was performed using Student's t‐test. *P < 0.05, **P < 0.01, and ***P < 0.001.

To systematically investigate the involvement of SIRTs in fibrotic bladder pathology, we performed high‐resolution single‐cell RNA sequencing (scRNA‐seq) on murine bladder tissues subjected to BOO induction. Following stringent quality control and doublet removal, a total of 55201 high‐quality cells were retained for downstream analysis (**Figure** **1**A). Unsupervised clustering identified five major cell populations, which were annotated based on canonical marker gene expression:^[^ ^27^, ^28^, ^29^, ^30^ ^]^ fibroblasts (*Pdgfra*), urothelial cells (UC, *Upk1a*), immune cells (IC, *Ptprc*), smooth muscle cells (SMC, *Acta2*), and Schwann cells (SC, *Cdh19*) (Figure 1A,B). The expression patterns of the top five marker genes for each cell cluster are visually summarized in Figure 1C. Gene Set Enrichment Analysis (GSEA) further indicated that elevated expression of fibroblast marker genes was significantly enriched in biological processes related to extracellular matrix (ECM) proteoglycans, ECM organization, and collagen formation (Figure 1D). Meanwhile, marker genes characterizing UC and IC were predominantly associated with keratinization and immune response pathways, respectively (Figure 1D). Furthermore, SMC and SC markers showed strong enrichment in terms related to muscle contraction and neuronal system development, respectively (Figure 1D). These findings collectively support the accuracy and biological relevance of our cell subpopulation classification and annotation.

Subsequent comparative analysis of SIRT family expression between control and fibrotic bladder tissues revealed that *Sirt1* was the only member exhibiting significant downregulation in the fibrotic group (Figures 1E; S1, Supporting Information). Immunofluorescence staining and Western blot analysis further indicated that SIRT1 is predominantly expressed within fibroblasts, and its expression was markedly reduced in these cells following BOO (Figure 1H–J). These observations were corroborated by bulk RNA sequencing data from a rat model of BOO, which demonstrated that DEGs in fibrotic bladders were enriched in pathways related to fibroblast proliferation, accompanied by decreased *Sirt1* expression (Figure 1F,G). To further validate our findings in vitro, we obtained and analyzed a transcriptomic dataset (GSE15871) from the Gene Expression Omnibus (GEO) database, which profiled the response of fibroblasts following TGF‐β1 stimulation. Consistent with our experimental observations in vivo, bioinformatic analysis revealed a significant downregulation of *Sirt1* expression in TGF‐β1‐treated fibroblasts compared to controls (Figure 1K). This reduction was further confirmed at the protein level through Western blot (Figure 1L). Collectively, these results consistently indicate that SIRT1 expression is suppressed in both fibrotic bladder tissues and activated fibroblasts. However, the precise molecular mechanisms and downstream pathways through which SIRT1 modulates fibroblast activation and extracellular matrix remodeling remain incompletely elucidated.

### 2.2 SIRT1‐FOXO3A‐BNIP3 Axis Inactivation Promotes Bladder Fibrosis via Impaired Mitophagy and Enhanced Oxidative Stress

**Figure 2:**
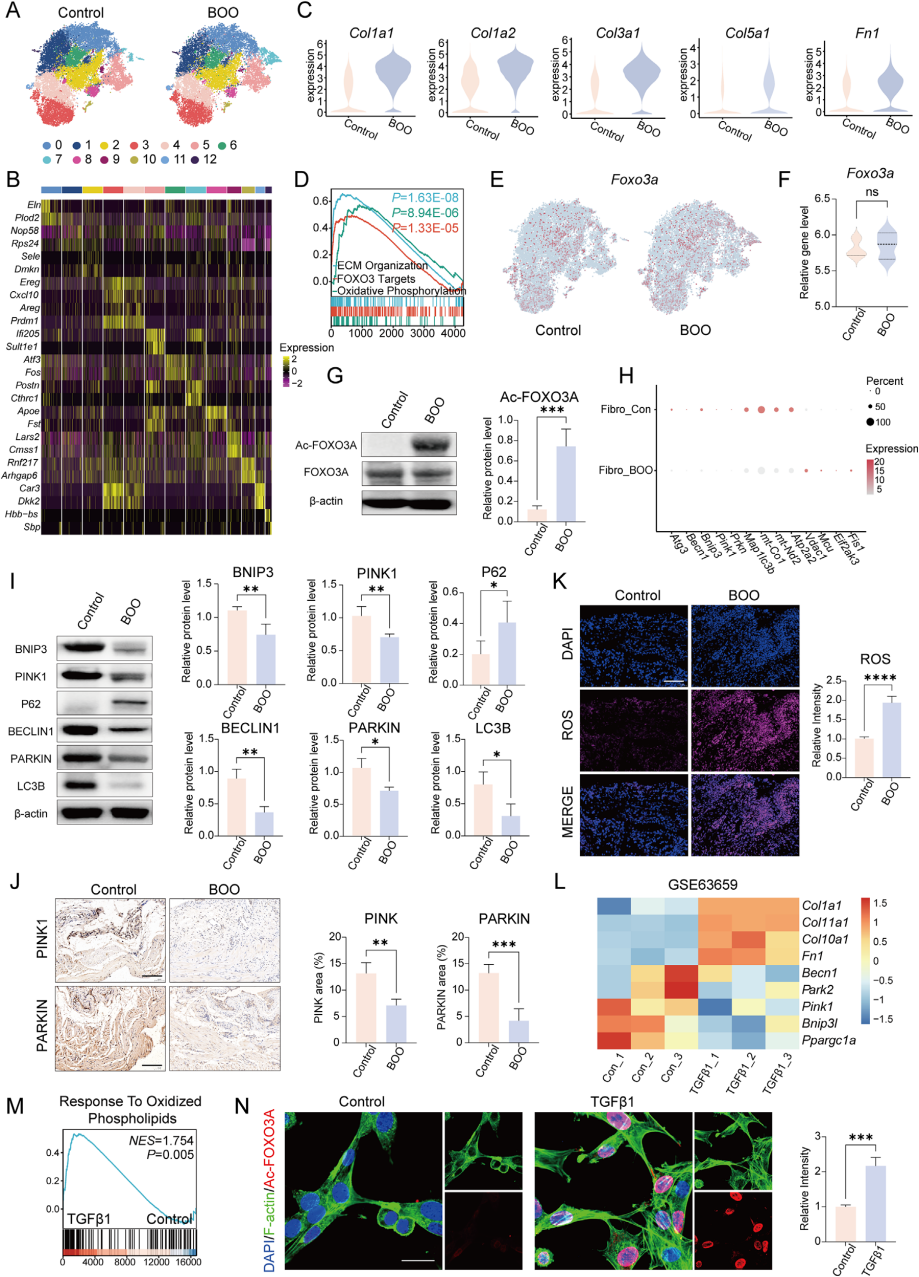
SIRT1‐FOXO3A‐BNIP3 Axis Inactivation Promotes Bladder Fibrosis via Impaired Mitophagy and Enhanced Oxidative Stress. A) t‐distributed stochastic neighbor embedding (t‐SNE) illustrating 13 transcriptionally distinct fibroblast subpopulations identified via re‐clustering analysis. B) Heatmap summarizing the top 3 marker genes in each cluster. C) Comparative transcriptomic profiling reveals significant upregulation of extracellular matrix (ECM)‐related genes, including *Col1a1*, *Col1a2*, *Col3a1*, *Col5a1*, and *Fn*, in fibroblasts isolated from fibrotic bladders relative to controls. D) Gene Set Enrichment Analysis (GSEA) of differentially expressed genes (DEGs) in fibrotic fibroblasts shows significant enrichment in biological processes associated with ECM organization, FOXO3A target genes, and oxidative phosphorylation. E) *Foxo3a* mRNA expression levels shows no significant differences across fibroblast subpopulations between groups. F) Bulk RNA sequencing data from a rat BOO model confirmed consistent *Foxo3a* transcript levels. G) Western blot analysis indicates a significant increase in FOXO3A acetylation in BOO‐induced bladders. Data are presented as the mean ± standard deviation (SD) (*n* = 3). H) Dot plot visualization of gene expression patterns related to mitochondrial function and quality control. I,J) Western blot and immunofluorescence analyses demonstrates downregulation of key mitophagy‐related proteins (PINK1, BECLIN1, PARKIN, LC3B) and accumulation of the autophagy adapter p62 in fibrotic bladders (scale bar = 100 µm). Data are presented as the mean ± SD (*n* = 3). K) BOO induction results in a substantial increase in oxidative stress (scale bar = 100 µm). Data are presented as the mean ± SD (*n* = 3). L,M) Integrated analysis of the publicly available dataset GSE15871 (Gene Expression Omnibus) confirms that TGF‐β1 stimulation significantly enhanced collagen secretion and oxidative stress in cultured fibroblasts. N) Immunofluorescence validation confirms increased FOXO3A acetylation in mouse bladder fibroblasts following TGF‐β1 treatment (scale bar = 25 µm). Data are presented as the mean ± SD (*n* = 3). Statistical analysis was performed using Student's *t*‐test. *P < 0.05, **P < 0.01, and ***P < 0.001.

To elucidate the precise molecular mechanisms and downstream signaling pathways by which SIRT1 regulates fibroblast activation, we isolated fibroblast and identified 13 distinct subpopulations based on unique marker gene expression patterns (**Figure** **2**A,B). Comparative transcriptomic analysis revealed significant upregulation of key ECM‐related genes, including *Col1a1*, *Col1a2*, *Col3a1*, *Col5a1*, and *Fn1*, in fibroblasts derived from fibrotic bladders compared to controls (Figure 2C). GSEA analysis further demonstrated that the upregulated DEGs in fibrotic fibroblasts were prominently enriched in biological processes such as ECM organization, FOXO3A targets, and oxidative phosphorylation (Figure 2D), suggesting a potential role for FOXO3A signaling and metabolic reprogramming in ECM remodeling. Notably, however, no significant difference in *Foxo3a* expression was detected between fibroblast subpopulations across groups (Figure 2E). This observation was corroborated by bulk RNA sequencing data from a rat model of BOO, which similarly showed consistent *Foxo3a* expression levels (Figure 2F).

Previous studies have established that SIRT1 modulates the subcellular localization and DNA‐binding capacity of FOXO3A via deacetylation, a key post‐translational modification.^[^ ^31^ ^]^ Consistent with this mechanism, we observed a significant increase in the acetylated state of FOXO3A following BOO (Figure 2G). This acetylated form of FOXO3A potentially functions as a transcriptional repressor of BNIP3, leading to impaired mitophagic flux and a subsequent rise in oxidative stress.^[^ ^32^ ^]^ To further elucidate the functional impact of this pathway under BOO conditions, we evaluated the expression of key mitophagy‐related proteins and assessed indicators of oxidative stress. Our results demonstrated that BOO induction led to marked dysregulation of mitophagy, concurrent with a substantial elevation in oxidative stress levels (Figure 2H–K). To further corroborate our experimental observations in vitro, we conducted a secondary analysis of a publicly available transcriptomic dataset (GSE15871). This analysis revealed that TGF‐β1 stimulation significantly promoted collagen secretion and elevated oxidative stress levels in cultured fibroblasts (Figure 2L,M). Consistent with these findings, we also detected a pronounced increase in FOXO3A acetylation following TGF‐β1 treatment in bladder fibroblasts (Figure 2N). Collectively, these results support the conclusion that inactivation of the SIRT1‐FOXO3A‐BNIP3 signaling axis, leading to impaired mitophagy and exacerbated oxidative stress, represents a central pathogenic mechanism underlying bladder fibrosis. Targeting this pathway represents a conceptually innovative and therapeutically promising approach to mitigate fibrotic pathology secondary to BOO.

### 2.3 Fabrication and Characterization of TSA

**Figure 3:**
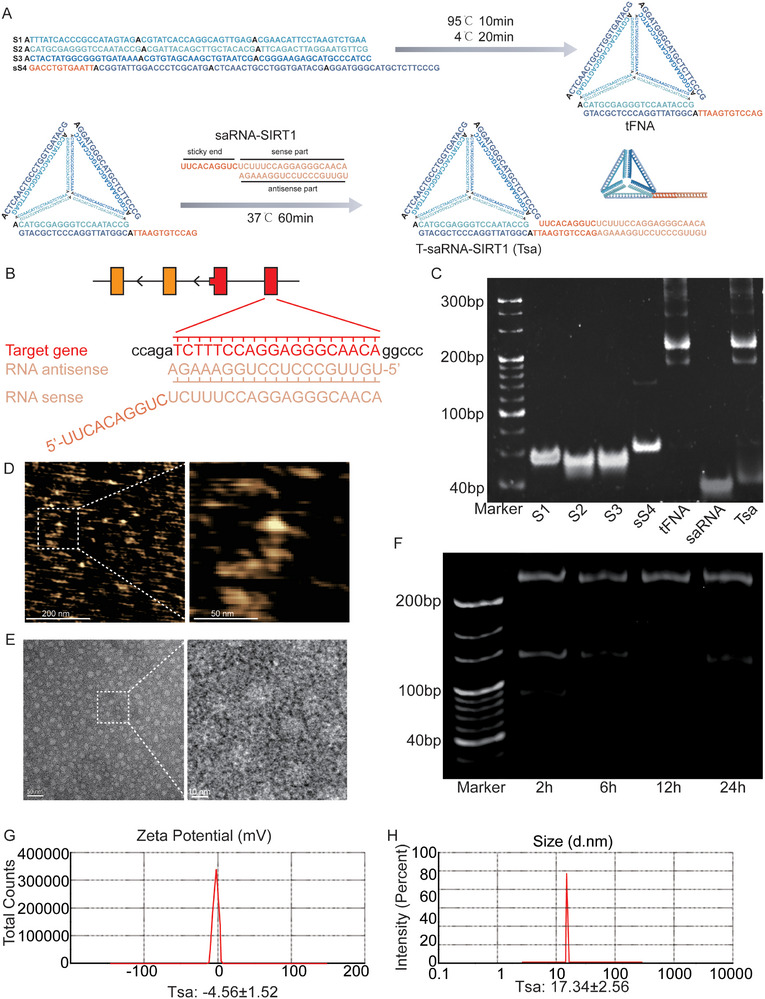
Fabrication and Characterization of TSA. A) Schematic diagram of assembling DNA and RNA nucleic acids into TSA. B) Illustration of saRNA antisense paired with transcriptional start site (TSS) +565 region of target gene SIRT1. C) Electrophoretic validation using 8% polyacrylamide gel (PAGE) confirming the successful two‐step synthesis of tFNA and TSA. Lanes (left to right): individual DNA strands (S1, S2, S3, sS4), assembled tFNA, saRNA, and final TSA complex. D,E) Structural morphology of TSA visualized via atomic force microscopy (AFM) and transmission electron microscopy (TEM). F) Remain of TSA in 10% FBS at 37 °C for 2, 6, 12, and 24 h. G,H) Dynamic light scattering detects the ζ‐potential and the particle size of TSA.

Based on the aforementioned findings, we hypothesize that therapeutic upregulation of SIRT1 expression could represent a promising strategy to mitigate BOO‐induced bladder dysfunction and fibrosis. In this context, small saRNA emerge as a potent epigenetic tool for targeted gene activation.^[^ ^33^ ^]^ However, the clinical translation of saRNAs is hampered by inherent limitations, including poor stability in serum, susceptibility to enzymatic degradation by RNases, and low cellular membrane permeability.^[^ ^14^ ^]^ To overcome these challenges, we designed and synthesized a functionalized tFNA conjugated with saRNA targeting the *Sirt1* (TSA). The TSA was fabricated through the self‐assembly of four single‐stranded DNA molecules and one double‐stranded RNA component (**Figure** **3**A). Transcriptional activation is initiated by the formation of the RITA complex, which is orchestrated by the saRNA guide strand upon its association with proteins such as AGO2, RHA, and CTR9.^[^ ^34^ ^]^ This complex then localizes to the SIRT1 gene promoter at a defined location of +565 bp relative to the transcriptional start site (TSS). The specificity of this interaction is ensured by the complete Watson‐Crick base pairing between the saRNA's antisense strand and the target DNA sequence (Figure 3B). The molecular weights of all constituent oligonucleotides (single‐stranded DNA and RNA), as well as the assembled tFNA and final TSA complex, were validated using 8% polyacrylamide gel electrophoresis (PAGE) (Figure 3C). Morphological examination was performed via atomic force microscopy (AFM) (Figure 3D) and transmission electron microscopy (TEM) (Figure 3E). To evaluate the protective efficacy of the TSA on its saRNA cargo, TSA was subjected to incubation in 10% fetal bovine serum over a series of time points (2, 6, 12, and 24 h) to quantitatively assess its degradation profile. The data indicated that within the serum environment, TSA effectively shielded the saRNA cargo from nucleolytic degradation and concurrently maintained a controlled, sustained release (Figure 3F). The physicochemical properties of TSA were characterized using dynamic light scattering (DLS), which revealed a hydrodynamic diameter of 17.34 ± 2.56 nm and a ζ‐potential of −4.56 ± 1.52 mV (Figure 3G). In summary, our findings confirm the successful assembly of TSA, a tetrahedral DNA functionalized with saRNA for targeted SIRT1 delivery.

### 2.4 Activation of SIRT1‐FOXO3A‐BNIP3 Axis by TSA Attenuates Fibroblast Activation and Oxidative Stress

**Figure 4:**
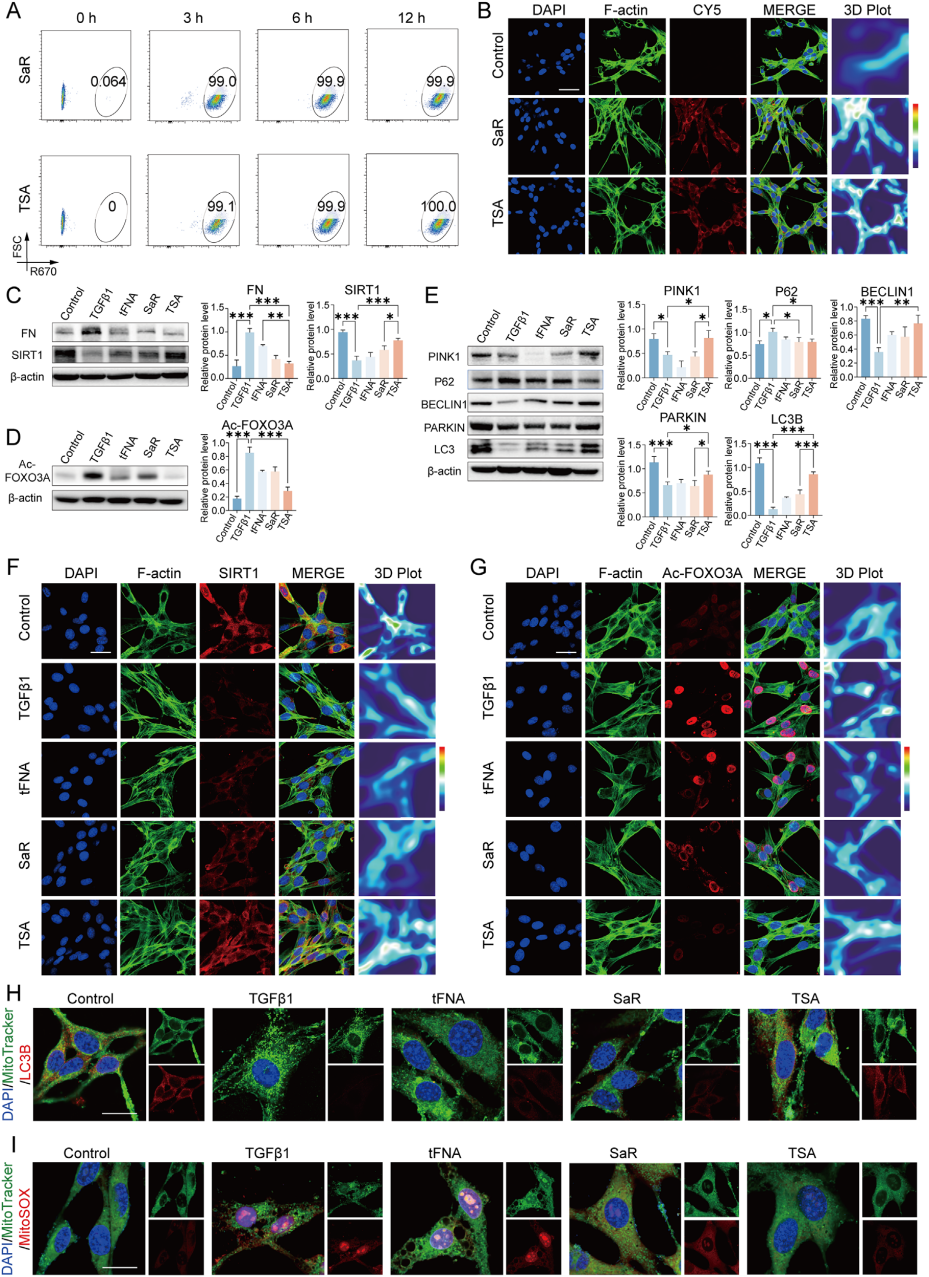
Activation of SIRT1‐FOXO3A‐BNIP3 Axis by TSA Attenuates Fibroblast Activation and Oxidative Stress. A) Cellular uptake efficiency of Cy5‐labeled SaR and TSA complexes in mouse bladder fibroblasts assessed at 3, 6, and 12 h post‐treatment. B) Immunofluorescence imaging confirming robust cytoplasmic internalization of SaR and TSA over a 12‐h incubation period (scale bar = 50 µm). C–E) Representative immunoblots and quantitative analysis of (C) SIRT1 and fibronectin (FN), (D) acetylation levels of FOXO3A, and (E) key mitophagy regulators (PINK1, BECLIN1, PARKIN, LC3) and autophagy adaptor P62 in cultured mouse bladder fibroblasts under varying treatment conditions. Data are presented as the mean ± standard deviation (SD) (*n* = 3). F,G) Representative immunostaining and thermal analysis of (F) SIRT1 and (G) FOXO3A acetylation in mouse bladder fibroblasts after different treatments (scale bar = 25 µm). H) Colocalization analysis of Mitotracker (mitochondria) and LC3B (autophagosomes) via immunofluorescence under different treatments (scale bar = 25 µm). I) Assessment of mitochondrial oxidative stress using Mitotracker and MitoSOX staining, visualized through fluorescence microscopy (scale bar = 25 µm). Statistical analysis was performed using one‐way analysis of variance (ANOVA) with Tukey's post hoc test for multiple comparisons. *P < 0.05, **P < 0.01, and ***P < 0.001.

To assess the cytoplasmic delivery efficiency of TSA, mouse bladder fibroblasts were incubated with Cy5‐labeled saRNA and Cy5‐conjugated TSA complexes for 3, 6, and 12 h. Flow cytometric analysis revealed that after a 3 h incubation period, over 99% of fibroblasts in both the SaR and TSA treatment groups exhibited positive fluorescence signals, with no statistically significant difference observed between the two groups (**Figure** **4**A). Furthermore, the mean fluorescence intensity demonstrated a time‐dependent increase, sustaining stable intracellular retention for up to 12 h (Figure 4A). Consistent with these findings, confocal laser scanning microscopy confirmed substantial cytoplasmic internalization of both SaR and TSA complexes throughout the 12‐h duration (Figure 4B), supporting efficient and prolonged cellular uptake. Subsequently, we assessed SIRT1 expression profiles across experimental groups and observed that TGF‐β1 stimulation significantly suppressed SIRT1 levels. This suppression was effectively reversed following treatment with both SaR and TSA, with TSA exhibiting the most pronounced restorative effect (Figure 4C,F). Consistent with SIRT1's role as a deacetylase, upregulation of SIRT1 via TSA administration markedly reduced the acetylation level of FOXO3A induced by TGF‐β1 (Figure 4D,G).

Given that acetylated FOXO3A transcriptionally represses *Bnip3* expression,^[^ ^32^ ^]^ a key regulator of mitophagy, we further evaluated mitophagy activity across different conditions. Mitophagy represents a selective autophagic process essential for maintaining mitochondrial quality control through the removal of damaged organelles, thereby attenuating ROS accumulation and inhibiting ECM remodeling. This process is regulated by two major pathways: the PINK1‐PARKIN axis and the BNIP3‐mediated pathway, which often exhibit crosstalk under pathological stimuli.^[^ ^35^ ^]^ Upon mitochondrial depolarization, PINK1 stabilizes on the outer mitochondrial membrane, recruiting and activating PARKIN to initiate autophagosome formation.^[^ ^35^ ^]^ Parallelly, BNIP3 functions as a mitophagy receptor that directly binds LC3 on the developing phagophore, facilitating the engulfment and lysosomal degradation of impaired mitochondria.^[^ ^35^ ^]^ Western blot analysis revealed that TGF‐β1 stimulation significantly downregulated the expression of key proteins essential for mitophagic initiation and execution, including PINK1, BECLIN1, PARKIN, and LC3B, while concurrently upregulating the autophagy adaptor protein p62 (Figure 4E). These alterations indicative of impaired mitochondrial clearance were markedly reversed by TSA administration, underscoring its potential as a positive regulator of mitochondrial quality control (Figure 4E,H). Furthermore, TGF‐β1 treatment notably elevated mtROS generation and fibronectin (Fn) secretion. This pathogenic induction was effectively suppressed upon TSA intervention (Figure 4C,I). Taken together, these findings indicate that TSA‐mediated SIRT1 activation facilitates FOXO3A deacetylation, thereby relieving its transcriptional repression of BNIP3. This molecular cascade attenuates TGF‐β1–induced mitochondrial dysfunction and mtROS overproduction, ultimately leading to the suppression of fibroblast activation and fibrotic progression.

### 2.5 TSA Attenuates BOO‐Induced Bladder Dysfunction and Fibrosis

**Figure 5:**
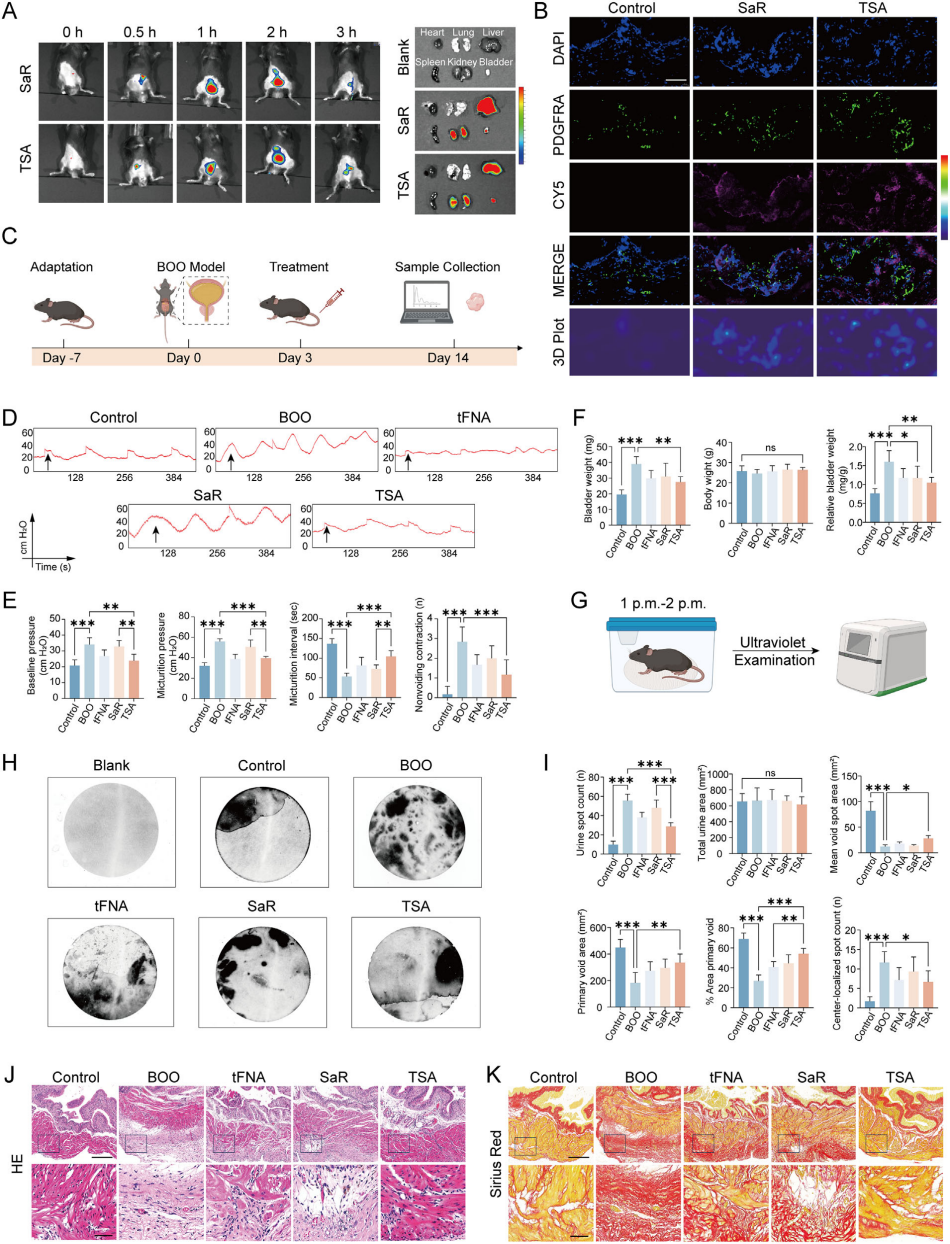
TSA Attenuates BOO‐Induced Bladder Dysfunction and Fibrosis. A) In vivo fluorescence imaging at 0.5, 1, 2, and 3 h post intravenous injection of Cy5‐labeled SaR and TSA complexes. Ex vivo imaging of major organs was performed at the 3‐h endpoint to evaluate biodistribution. B) Immunofluorescence staining of bladder sections showing successful co‐localization of SaRNA and TSA with PDGFRA‐positive fibroblasts (scale bar = 50 µm). C) Schematic overview of the experimental timeline and treatment protocol in the BOO‐induced murine model. D) Representative urodynamic tracings depicting intravesical pressure changes during filling and voiding phases; Black arrow indicates a single voiding event. E,F) Quantitative analyses of urodynamic parameters: (E) baseline pressure, micturition pressure, micturition interval, non‐voiding contraction; (F) bladder weight, body weight, relative bladder weight across experimental groups. Data are presented as the mean ± standard deviation (SD) (*n* = 4) G) Schematic illustration of void spot assay. H,I) Representative images and analyses from voiding spot assay among five groups. Data are presented as the mean ± SD (*n* = 4) J,K) Representative histopathological images of bladder sections stained with (J) hematoxylin and eosin (H&E) and (K) Sirius red among five groups (scale bar = 200 or 50 µm). Statistical analysis was performed using one‐way analysis of variance (ANOVA) with Tukey's post hoc test for multiple comparisons. *P < 0.05, **P < 0.01, and ***P < 0.001.

To assess the tissue penetration and cellular uptake efficiency of TSA within the bladder, both saRNA and the TSA complex were conjugated with the near‐infrared fluorophore Cy5. Real‐time in vivo fluorescence imaging revealed a time‐dependent change in fluorescence signal intensity: an initial gradual increase was observed within the first 2 h, followed by a progressive decline in both the SaRNA and TSA treatment groups (**Figure** **5**A). At the 3 h time point, all animals were euthanized, and major organs, including the heart, lungs, liver, spleen, kidneys, and bladder were harvested for *ex vivo* imaging. Notably, the bladder exhibited sustained and pronounced Cy5‐derived fluorescence in both the SaRNA and TSA groups, indicating effective local accumulation and retention (Figure 5A). We further investigated the cellular internalization of SaRNA and TSA within bladder fibroblasts. Immunofluorescence staining of bladder tissue sections demonstrated robust co‐localization of both SaRNA and TSA signals with PDGFRA‐positive fibroblasts (Figure 5B), confirming successful delivery into target cells and supporting their subsequent engagement in downstream biological processes.

To evaluate the therapeutic potential of TSA on bladder dysfunction and fibrosis, we established a BOO model in 40 male mice using a previously validated surgical protocol.^[^ ^36^, ^37^, ^38^ ^]^ Beginning on the third postoperative day, the mice received tail‐vein injections of tFNA, SaR, or TSA (100 µL per administration at specified concentrations) every 48 h. Two weeks following surgery, bladder tissues were carefully harvested for subsequent analysis (Figure 5C). Urodynamic assessment revealed a significant elevation in both baseline and maximum voiding pressures in the BOO group compared to sham‐operated controls. TSA treatment markedly attenuated these BOO‐induced increases in voiding pressures (Figure 5D,E). Since a reduced micturition interval is an established early indicator of BOO, we compared micturition intervals among the experimental groups and found that TSA administration significantly prolonged the urinary interval compared to the BOO group (Figure 5D,E). Furthermore, TSA treatment substantially reduced the frequency of non‐voiding contractions relative to the BOO group (Figure 5D,E). No significant differences in body weight were observed across the five groups. However, TSA administration significantly suppressed BOO‐induced bladder hypertrophy, as evidenced by a reduction in relative bladder weight (Figure 5F).

To further evaluate voiding function across experimental groups, we employed the void spot assay (Figure 5G), a well‐established, non‐invasive technique for quantifying murine urinary behavior.^[^ ^39^ ^]^ Mice subjected to BOO exhibited a significant increase in urine spot count compared to sham controls (Figure 5H,I), consistent with elevated voiding frequency. This pathological pattern was notably ameliorated following treatment with tFNA, SaR, or TSA, with the most substantial improvement observed in the TSA group (Figure 5H,I). Total urine area, however, remained comparable across all groups, indicating that neither BOO induction nor nanomaterial administration significantly influenced overall urinary output (Figure 5H,I). Quantitative assessment of voiding patterns revealed a reduction in mean void spot area, primary void area (largest single void event), and the proportion of primary void area in BOO mice relative to controls, findings indicative of urinary frequency and urgency.^[^ ^40^ ^]^ Intervention with tFNA or SaR partially restored these parameters, while TSA treatment produced the most pronounced restorative effect (Figure 5H,I). Spatial mapping of voiding events showed that control mice primarily urinated at the periphery or corners of the filter paper, whereas BOO animals exhibited dispersed voiding patterns with increased central localization, suggesting urinary incontinence.^[^ ^41^ ^]^ TSA administration significantly reduced the number of centrally localized spots (Figure 5H,I).

To assess fibrotic remodeling, bladder sections from all five groups were subjected to hematoxylin and eosin (H&E) staining. BOO‐induced thickening of the lamina propria was markedly attenuated by TSA treatment (Figure 5J). Sirius red staining further revealed increased collagen deposition (red staining) in obstructed bladders, which was substantially suppressed by TSA administration (Figure 5K). These collective findings demonstrate that TSA effectively alleviates BOO‐induced dysfunction and structural fibrosis.

### 2.6 Restoration of SIRT1‐FOXO3A‐BNIP3 Axis by TSA Mitigates Oxidative Stress in BOO Model

**Figure 6:**
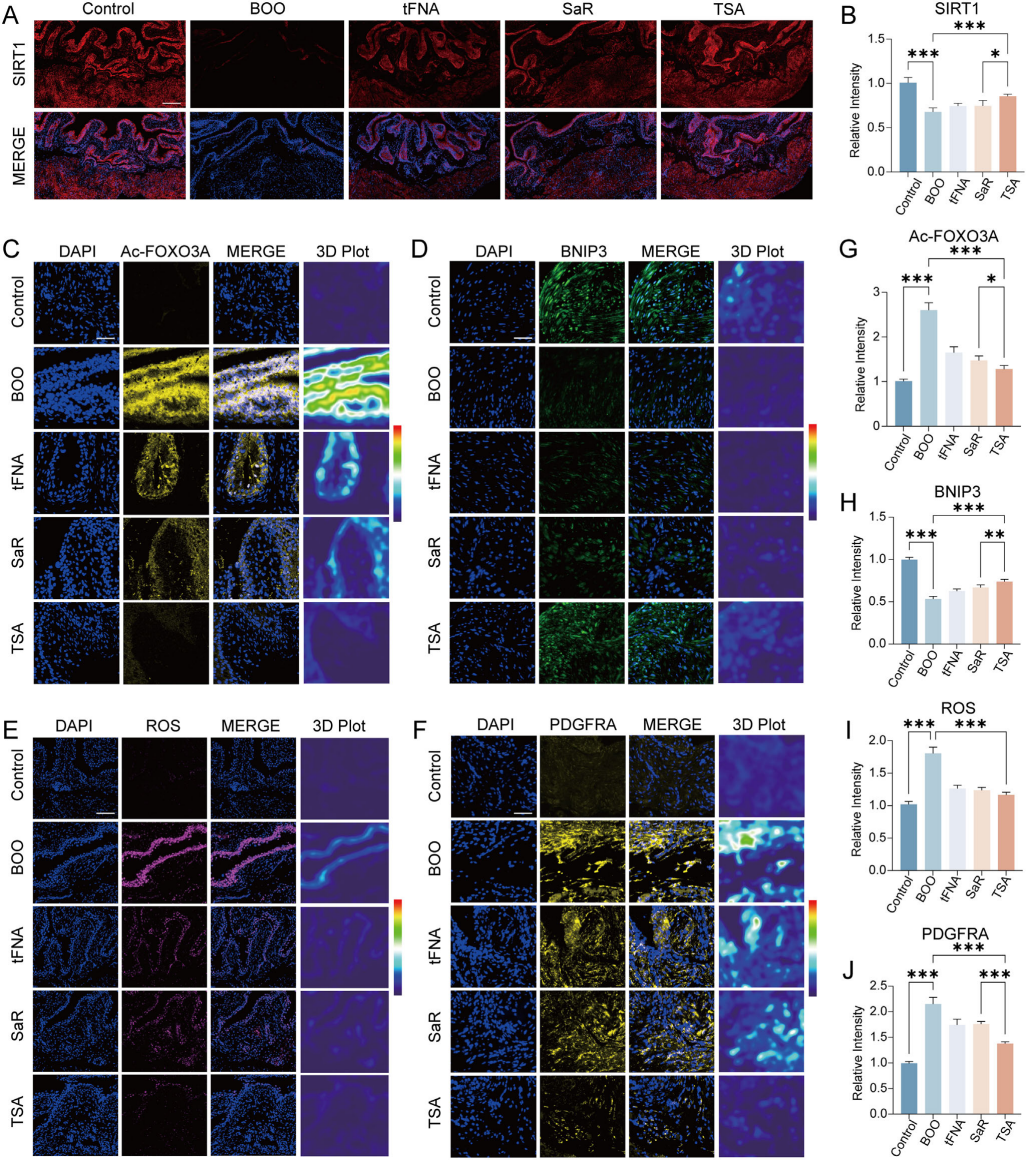
Restoration of SIRT1‐FOXO3‐BNIP3 Axis by TSA Mitigates Oxidative Stress in BOO Model. A,B) Immunofluorescence staining and corresponding statistical analysis of SIRT1 across the five groups (scale bar = 200 µm). Data are presented as the mean ± standard deviation (SD) (*n* = 4). C–F) Representative immunofluorescence images depicting (C) acetylated FOXO3A, (D) BNIP3, (E) reactive oxygen species (ROS), and (F) PDGFRA (a fibroblast marker) in bladder tissues from each group (scale bar = 50 µm). G–J) Quantitative evaluation of fluorescence intensity for (G) FOXO3A acetylation, (H) BNIP3 expression, (I) ROS accumulation, and (J) PDGFRA⁺ fibroblast presence across all groups. Data are presented as the mean ± SD (*n* = 4). Statistical analysis was performed using one‐way analysis of variance (ANOVA) with Tukey's post hoc test for multiple comparisons. *P < 0.05, **P < 0.01, and ***P < 0.001.

Given the observed protective effects of TSA against bladder dysfunction and fibrotic remodeling, we next evaluated the expression profiles of key components within the SIRT1‐FOXO3A‐BNIP3 signaling axis across five experimental groups. Immunofluorescence staining revealed a significant downregulation of SIRT1 expression following BOO induction. This suppression was effectively rescued by both SaR and TSA treatment, with TSA exhibiting the most robust restorative capacity (**Figure** **6**A,B). Furthermore, TSA‐mediated upregulation of SIRT1 markedly reduced the BOO‐induced hyperacetylation of FOXO3A (Figure 6C,G). Concomitantly, BNIP3, a downstream transcriptional target of acetylated FOXO3A, was significantly downregulated in BOO‐induced bladders, indicating impaired mitophagy. Administration of TSA substantially reversed these alterations and restored BNIP3 expression (Figure 6D,H). In line with these molecular changes, BOO induction triggered substantial ROS accumulation (Figure 6E,I) and fibroblast activation (Figure 6F,J), both of which were significantly attenuated by TSA treatment. Finally, to evaluate the biosafety profile of the administered nanomaterials, major organs (heart, lung, liver, spleen, and kidney) were harvested from each experimental group and examined via H&E staining (Figure S2, Supporting Information). No significant histopathological abnormalities were observed in any treatment group, confirming the in vivo biocompatibility and safety of tFNA, SaR, and TSA.

## 3 Conclusion

**Figure 7:**
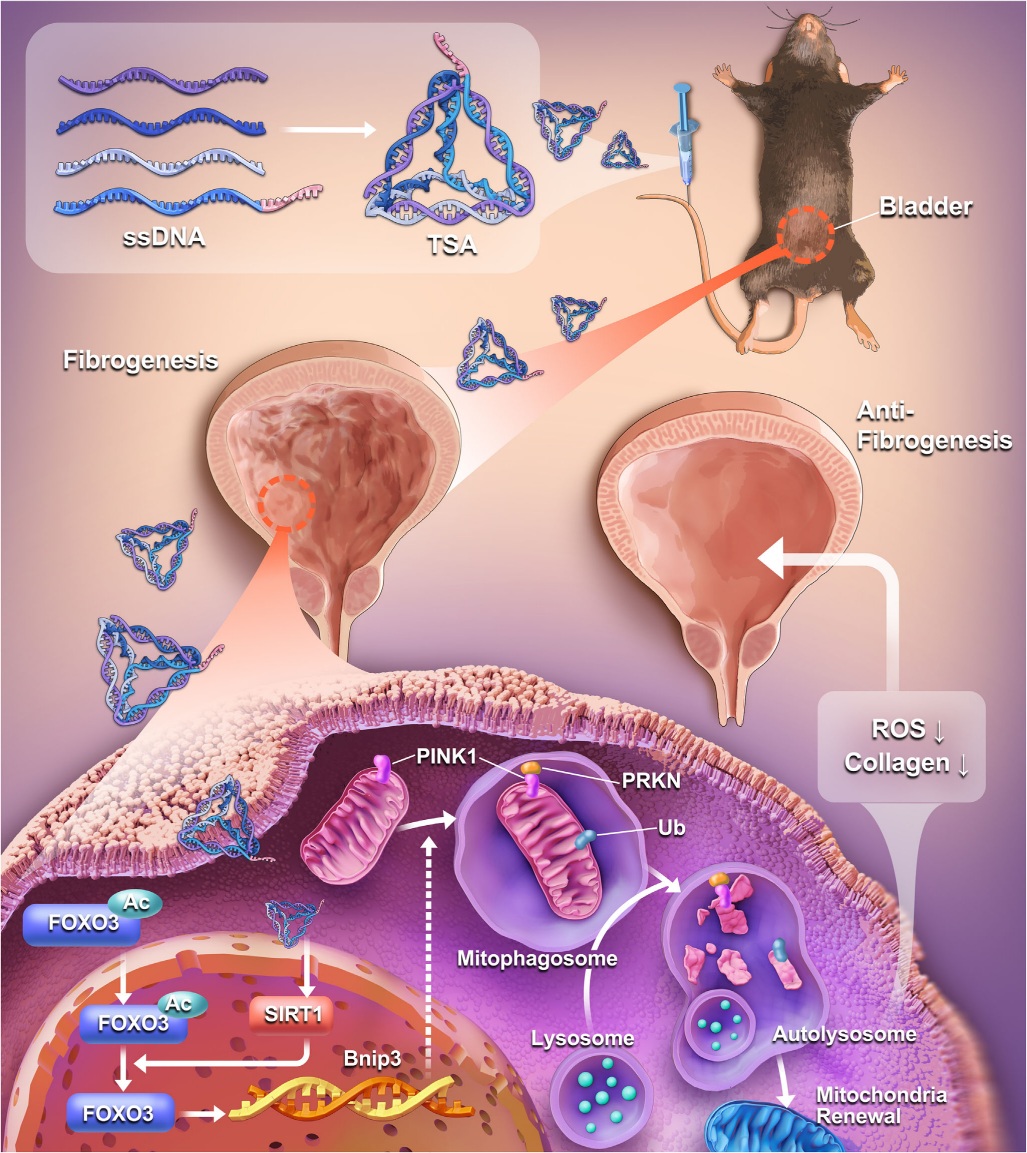
A proposed mechanism of the novel SIRT1‐targeted saRNA‐delivering tetrahedral DNA (TSA) in mitigating bladder fibrosis. The TSA treatment effectively upregulates SIRT1 expression, which subsequently promotes FOXO3A deacetylation. This deacetylation event relieves FOXO3A's transcriptional repression on the BNIP3 gene, thereby initiating PINK1‐PARKIN‐dependent mitophagy. The enhancement of mitophagic flux ultimately reduces mitochondrial ROS accumulation, resulting in the inhibition of fibroblast activation and pathological collagen deposition.

In summary, through an integrated analysis of single‐cell and bulk transcriptomic data, our study identified SIRT1 as the sole member of the sirtuin family exhibiting significant downregulation in fibrotic bladder tissues and activated fibroblasts. To address this dysregulated pathway, we developed TSA, a novel tetrahedral DNA engineered for targeted SIRT1 activation. This system demonstrates exceptional biocompatibility, enhanced cellular permeability, and superior RNA cargo protection, effectively overcoming the limitations associated with standalone saRNA therapeutics. Treatment with TSA robustly restored SIRT1 expression, which facilitated FOXO3A deacetylation and alleviated its transcriptional repression of BNIP3. This cascade led to the activation of PINK1‐PARKIN‐mediated mitophagy and reduced mtROS overproduction, and ultimately inhibited fibroblast activation and collagen deposition (**Figure** **7**). Our findings establish a targeted, tetrahedral DNA‐based gene activation strategy as a promising therapeutic intervention for bladder fibrosis, with significant potential for future clinical translation.

## 4 Experimental Section

### Synthesis of TSA

The TSA was fabricated via a two‐step assembly process. Initially, four single‐stranded DNA sequences (S1, S2, S3, and S4; Table S1, Supporting Information) were combined in equimolar ratios in TM buffer (10 mm Tris‐HCl, 50 mm MgCl_2_·6H_2_O, pH 8.0).^[^ ^42^, ^43^, ^44^, ^45^, ^46^ ^]^ The mixture was subsequently subjected to thermal annealing at 95 °C for 10 min, followed by rapid cooling to 4 °C for 20 min to facilitate the self‐assembly of DNA into tFNAs via Watson–Crick base pairing.^[^ ^24^, ^47^, ^48^ ^]^ Finally, the tFNAs were incubated with an equimolar concentration of saRNA‐SIRT1 at 37 °C for 1 h to form the final TSA complex.

### TSA Characterization

To confirm the successful formation of TSA, non‐denaturing polyacrylamide gel electrophoresis (PAGE; 90 V, 60 min) and high‐performance capillary electrophoresis (HPCE) were employed. The hydrodynamic diameter and surface charge (ζ‐potential) of the nanostructures were characterized using dynamic light scattering (DLS; Malvern Nano ZS, UK). Morphological examination was conducted using atomic force microscopy (AFM; Shimadzu SPM‐9700, Japan) and transmission electron microscopy (TEM; Zeiss Libra200, Germany). Furthermore, the structural integrity of TSA was evaluated under physiological conditions by incubating both TSA and naked saRNA (1000 nm) in 10% fetal bovine serum at 37 °C for time intervals of 0, 4, 8, 12, and 24 h. The degradation profiles were subsequently assessed using 2% agarose gel electrophoresis. To assess the cytoplasmic delivery efficiency of TSA, mouse bladder fibroblasts were incubated with Cy5‐labeled saRNA and Cy5‐conjugated TSA complexes. Cellular internalization was evaluated at 3, 6, and 12 h post‐treatment. Following incubation, cells were fixed in 4% paraformaldehyde, permeabilized with Triton X‐100, and counterstained with DAPI. Fluorescence imaging was performed using a high‐resolution confocal microscope (TiA1‐N‐STORM, Nikon, Japan).

### Cell Treatment

Mouse bladder fibroblasts (PRI‐MOU‐00118; Shanghai Zhong Qiao Xin Zhou Biotechnology Co., Ltd) were maintained in Mouse Bladder Fibroblast Complete Medium (PCM‐M‐118; Shanghai Zhong Qiao Xin Zhou Biotechnology Co., Ltd) at 37 °C under a 5% CO_2_ atmosphere with saturated humidity. For subsequent interventions, cells were allocated into five experimental groups. With the exception of the control group, which received no additional treatment, the remaining four groups were stimulated with 5 ng·mL^−1^ recombinant TGF‐β1(PeproTech) for 24 h.^[^ ^38^, ^49^ ^]^ Concurrently, tFNAs, SaR, and TSA were administered to their respective groups at a concentration of 250 nm.^[^ ^38^, ^49^ ^]^

### Animal Models

All animal experiments conducted in this study were reviewed and approved by the Experimental Animal Ethics Committee of West China Hospital, Sichuan University. The procedures strictly adhered to the principles outlined in the Declaration of Helsinki for the use of animals in research. Male C57BL/6 mice (8 weeks old, 20–25 g body weight) were sourced from GemPharmatech Co., Ltd. (Nanjing, China). A BOO model was established following a previously described surgical protocol.^[^ ^36^, ^37^, ^38^ ^]^ Briefly, after anesthesia induction, mice were positioned supine on a thermostatic heating pad maintained at 37 °C. A midline laparotomy was performed to expose the bladder and proximal urethra. Partial BOO was induced by ligating the proximal urethra using a 6–0 nylon suture. A 24‐gauge polyethylene rod was placed adjacent to the urethra during ligation to standardize the degree of obstruction and prevent complete occlusion. The rod was subsequently withdrawn, and the abdominal incision was closed in layers. Sham‐operated control mice underwent identical surgical procedures except for urethral ligation.

A total of 40 male C57BL/6 mice were randomly allocated into five experimental groups (*n* = 8 per group) as follows: 1) Control group (sham‐operated); 2) BOO group; 3) BOO + tFNAs group (250 nm); 4) BOO + SaR group (250 nm); and 5) BOO + TSA group (250 nm). Beginning on the third postoperative day, mice in groups (3–5) received tail vein injections of the respective agents (100 µL per injection) every 48 h. Two weeks following the BOO surgery, all mice were euthanized. Bladders were then carefully harvested, thoroughly emptied of residual urine, and weighed for subsequent analysis

### Urodynamics

Urodynamic assessment was performed on post‐operative day 14 prior to tissue harvest to evaluate functional alterations following BOO. Mice were anesthetized with isoflurane and placed in a supine position. A midline laparotomy was conducted to expose the urinary bladder, and a polyethylene catheter was implanted into the bladder dome via a small cystostomy. The catheter was secured with a 6‐0 silk purse‐string suture to prevent leakage and connected to a dual‐channel pressure transducer (BL‐420N, Tai Meng Technology Co., Ltd., Chengdu, China) and a micro‐infusion pump. After complete bladder emptying, continuous filling cystometry was initiated by infusing warm sterile saline (37 °C) at a constant rate of 10 µL min^−1^.^[^ ^38^ ^]^ Following a stabilization period of ≈30 min, 6–10 consecutive micturition cycles were recorded for each animal. A micturition cycle was defined as the interval between the return of intravesical pressure to baseline following one voiding event and its subsequent return to baseline after the next void. The maximum intravesical pressure during each voiding event was recorded quantified. Non‐voiding contractions were identified as spontaneous, phasic increases in detrusor pressure (amplitude ≥ 4 cm H_2_O from baseline) occurring during the filling phase without concomitant urine outflow.^[^ ^38^ ^]^

### Void Spot Assay

To evaluate voluntary micturition patterns in conscious mice, a void spot assay was performed following established protocols.^[^ ^39^ ^]^ Briefly, each mouse was individually housed in a clean cage lined with filter paper (Whatman Grade 540, 125 mm diameter; Waltham, Cat. #1540‐125) for a duration of 1 h (13:00‐14:00). Food was provided but water was withheld to avoid potential confounders related to fluid intake. Following the assay, the filter paper was carefully collected and visualized under ultraviolet illumination to enhance the contrast of urine spots. Image acquisition and quantitative analysis were conducted using ImageJ software (v1.54p). To minimize artifacts caused by non‐urine deposits (e.g., scratches or moisture from paws and tails), all spots with an area less than 0.02 cm^2^ were excluded from analysis.^[^ ^41^ ^]^ Several key voiding parameters were quantified: 1) Urine spot count: number of urine spots ≥ 0.02 cm^2^; 2) Total urine area: estimated from cumulative urine spot area; 3) mean void area: (total urine area) ÷ (urine count number); 4) Primary void area: defined as the single largest spot per mouse, reflecting maximal voiding effort;^[^ ^40^ ^]^ 5) Primary void ratio: percentage of total voided area accounted for by the primary void (primary void area ÷ total area × 100); 6) Central‐localized spot count: number of spots located within the central 50% of the filter paper, a potential indicator of overactive bladder or loss of voluntary control.^[^ ^41^ ^]^

### Live Animal Imaging System

To evaluate the in vivo biodistribution and pharmacokinetic profile of the administered agents, near‐infrared fluorescence imaging was performed using a live animal imaging system (IVIS Spectrum, PerkinElmer, USA) following established protocols.^[^ ^39^ ^]^ Prior to intravenous injection, the lower abdominal region of each mouse was carefully shaved to minimize interference. Subsequently, 100 µL of Cy5‐labeled SaR (250 nm) or Cy5‐labeled TSA (250 nm) was administered via tail vein injection. Mice were anesthetized with isoflurane and placed in a prone position within the imaging chamber. Whole‐body fluorescence was captured at 0.5, 1, 2, and 3 h post‐injection. A sham‐injected control group (no fluorophore) was included to account for background autofluorescence and non‐specific signals. After the final imaging time point (3 h), all animals were euthanized, and major organs‐including the heart, lungs, liver, spleen, kidneys, and bladder‐were harvested for ex vivo fluorescence imaging to quantify regional accumulation.

### Histopathological Analysis

Following euthanasia, bladder tissues along with five major organs (lungs, heart, liver, spleen, and kidneys) were harvested and immediately fixed in 10% neutral buffered formalin. The samples were subsequently dehydrated through a graded ethanol series, embedded in paraffin, and sectioned into 4‐µm slices using a microtome. For histological evaluation, sections from both bladder and other organs were subjected to H&E staining. All stained sections were scanned using a high‐resolution digital slide scanner (Pannoramic MIDI; 3DHISTECH, Hungary) for subsequent imaging and morphological analysis.

Intracellular reactive oxygen species (ROS) levels were detected across experimental groups using a commercial ROS assay kit (Beyotime, Cat. No. S0033S) according to the manufacturer's instructions. Furthermore, immunohistochemical staining was performed to assess the protein expression levels of PINK1 (ABclonal, #A7131) and PARKIN (ABclonal, #A0968). All IHC‐stained sections were similarly scanned and digitized using the same tissue scanning system for consistent quantitative and qualitative analysis.

### Immunofluorescence Staining

The samples were fixed with 4% paraformaldehyde for 20 min at room temperature. Permeabilization was then carried out using 0.5% Triton X‐100, followed by blocking with 5% normal goat serum to prevent nonspecific binding. For immunofluorescence staining, the samples were incubated overnight at 4 °C with the following primary antibodies: anti‐SIRT1 (Affinity, #DF6033), anti‐acetyl‐FOXO3A (Affinity, #AF3771), anti‐LC3B (Affinity, #AF4650), anti‐PDGFRA (Affinity, #AF0241), and anti‐BNIP3 (ABclonal, #A5683). After thorough washing, the cells were incubated with species‐appropriate fluorescently conjugated secondary antibodies. Nuclei were counterstained with 4′,6‐diamidino‐2‐phenylindole (DAPI). Finally, high‐resolution images were acquired using a confocal microscope (Nikon TiA1‐N‐STORM, Japan) under consistent imaging parameters

### Western Blotting

A commercial whole protein extraction kit (KeyGen BioTECH, Cat. #KGB5300‐50) was utilized to extract total cellular proteins, according to the manufacturer's instructions. The extracted protein samples were subsequently subjected to ultracentrifugation and ultrasonication to ensure complete homogenization and solubility. Thereafter, protein suspensions were mixed with 5× protein loading buffer at a 4:1 volume ratio and denatured by boiling at 95 °C for 10 min. Protein expression levels were quantified following well‐established protocols. The following primary antibodies were employed at a dilution of 1:1000: anti‐acetyl‐FOXO3A (Affinity, #AF3771), anti‐FOXO3A (Affinity, #AF6020), anti‐BNIP3 (ABclonal, #A5683), anti‐PINK1 (ABclonal, #A7131), anti‐p62 (Affinity, #AF5384), anti‐BECLIN1 (Affinity, #AF5128), anti‐PARKIN (ABclonal, #A0968), anti‐LC3B (Affinity, #AF4650), anti‐SIRT1 (Affinity, #DF6033), and anti‐LC3 (Affinity, #AF5402). Anti‐β‐Actin (ABclonal, #AC026) was used as an internal loading control. After incubation with species‐appropriate horseradish peroxidase (HRP)‐conjugated secondary antibodies, immunoreactive signals were visualized using a chemiluminescence imaging system (ChemiDoc MP, Bio‐Rad, USA).

### ScRNA‐Seq Analysis

The single‐cell transcriptomic analysis was performed using the SeekOne MM (Micwell & Magnetic Beads) platform. Total RNA was extracted from mouse bladder tissues obtained from both control and BOO groups (*n* = 3 per group) with TRIzol reagent. Sequencing was carried out on either an Illumina HiSeq XTen or NovaSeq system. Raw sequencing reads were processed with fastp to remove primer sequences and low‐quality bases, and to generate basic sequencing statistics. Downstream bioinformatic analysis included clustering and visualization of single‐cell data using the Seurat package. Cell type annotation was conducted by referencing curated marker gene sets within the SingleR framework. To elucidate biological functions and pathway activities, Gene Ontology (GO) and Kyoto Encyclopedia of Genes and Genomes (KEGG) enrichment analyses were performed on cluster‐specific differentially expressed genes (DEGs) using the ClusterProfiler tool. Genes meeting the threshold of |log_2_(fold change)| ≥ 1.5 and an adjusted *P*‐value < 0.05 were considered statistically significant.

### Statistical Analysis

Statistical analysis was performed using Student's *t*‐test and one‐way analysis of variance (ANOVA) with Tukey's post hoc test for multiple comparisons to control the family‐wise error rate. All computations and graphical representations were generated using GraphPad Prism (Version 10.4.0; GraphPad Software Inc., San Diego, CA, USA). Quantitative data obtained from at least three independent experiments are presented as mean ± standard deviation (SD). Error bars in graphical figures correspond to SD. Differences were considered statistically significant at *P* < 0.05.

## Conflict of Interest

The authors declare no conflict of interest.

## Supporting information



Supporting Information

## Data Availability

The data that support the findings of this study are available from the corresponding author upon reasonable request.
