# Supplementary material for: A Sirtuin‐1‐Targeted Gene‐Activating Tetrahedral DNA Attenuates Bladder Fibrosis by Restoring Mitophagy in Fibroblasts via the SIRT1‐FOXO3‐BNIP3 Axis
Source: Adv Sci (Weinh). 2025 Nov 23;13(8):e19527. doi: 10.1002/advs.202519527 (PMC12884717; doi:10.1002/advs.202519527)
Supplement: Supplementary file 1 — Supporting Information [file ADVS-13-e19527-s001.docx]

**Supporting Information**

**Title:** A Sirtuin-1-Targeted Gene-Activating Tetrahedral DNA Attenuates Bladder Fibrosis by Restoring Mitophagy in Fibroblasts via the SIRT1-FOXO3-BNIP3 Axis​

**Running title:** Sirtuin-1-Targeted Tetrahedral DNA Attenuates Bladder Fibrosis

**Authors:** *Wei Wang****^#^****^1^, Ran Yan****^#^****^2^, Lede Lin****^#^****^1^, Lei Xiang^1^, Xiaozhi Xia^1^, Liao Peng^1^, Xiaoshuai Gao^1^, Jiawei Chen^1^**, Yang Xiong^1^, Liang Zhou*^1^, Yunfeng Lin*^2^*

**Authors affiliation:**

^1^ Department of Urology, Institute of Urology (Laboratory of Reconstructive Urology), West China Hospital, Sichuan University, Chengdu, Sichuan, P.R. China.

^2^ State Key Laboratory of Oral Diseases, National Clinical Research Center for Oral Diseases, West China Hospital of Stomatology, Sichuan University, Chengdu, 610041, China.

***. Corresponding author:**

**Liang Zhou:** Department of Urology, Institute of Urology (Laboratory of Reconstructive Urology), West China Hospital, Sichuan University, No. 37 Guo Xue Xiang, Chengdu, Sichuan, 610041, P.R. China. **E-mail:** [zhouliang5678@foxmail.com](mailto:zhouliang5678@foxmail.com)

**Yunfeng Lin:** State Key Laboratory of Oral Diseases, National Clinical Research Center for Oral Diseases, West China Hospital of Stomatology, Sichuan University, Chengdu, 610041, China. **E-mail:** [yunfenglin@scu.edu.cn](mailto:yunfenglin@scu.edu.cn)

**Supporting Information:**

Table S1

Figure S1-S2

**Supplementary Table S1.** Sequence of Four Designed ssDNAs with Sticker and Designed saRNA-SIRT1.

| ssDNA | Sequence (5′-3′) |
| --- | --- |
| S1 | ATTTATCACCCGCCATAGTAGACGTATCACCAGGCAGTTGA GACGAACATTCCTAAGTCTGAA |
| S2 | ACATGCGAGGGTCCAATACCGACGATTACAGCTTGCTACAC GATTCAGACTTAGGAATGTTCG |
| S3 | ACTACTATGGCGGGTGATAAAACGTGTAGCAAGCTGTAATC GACGGGAAGAGCATGCCCATCC |
| S4 | ACGGTATTGGACCCTCGCATGACTCAACTGCCTGGTGATAC GAGGATGGGCATGCTCTTCCCG |
| Sticky end | GACCTGTGAATT |
| sS4 | GACCTGTGAATTACGGTATTGGACCCTCGCATGACTCAACT GCCTGGTGATACGAGGATGGGCATGCTCTTCCCG |
| SaRNA-SIRT1 (sense) | UUCACAGGUCUCUUUCCAGGAGGGCAACA |
| SaRNA-SIRT1 (antisense) | UGUUGCCCUCCUGGAAAGA |
| SaRNA-SIRT1 (antisense)-Cy5 | UGUUGCCCUCCUGGAAAGA -Cy5 |


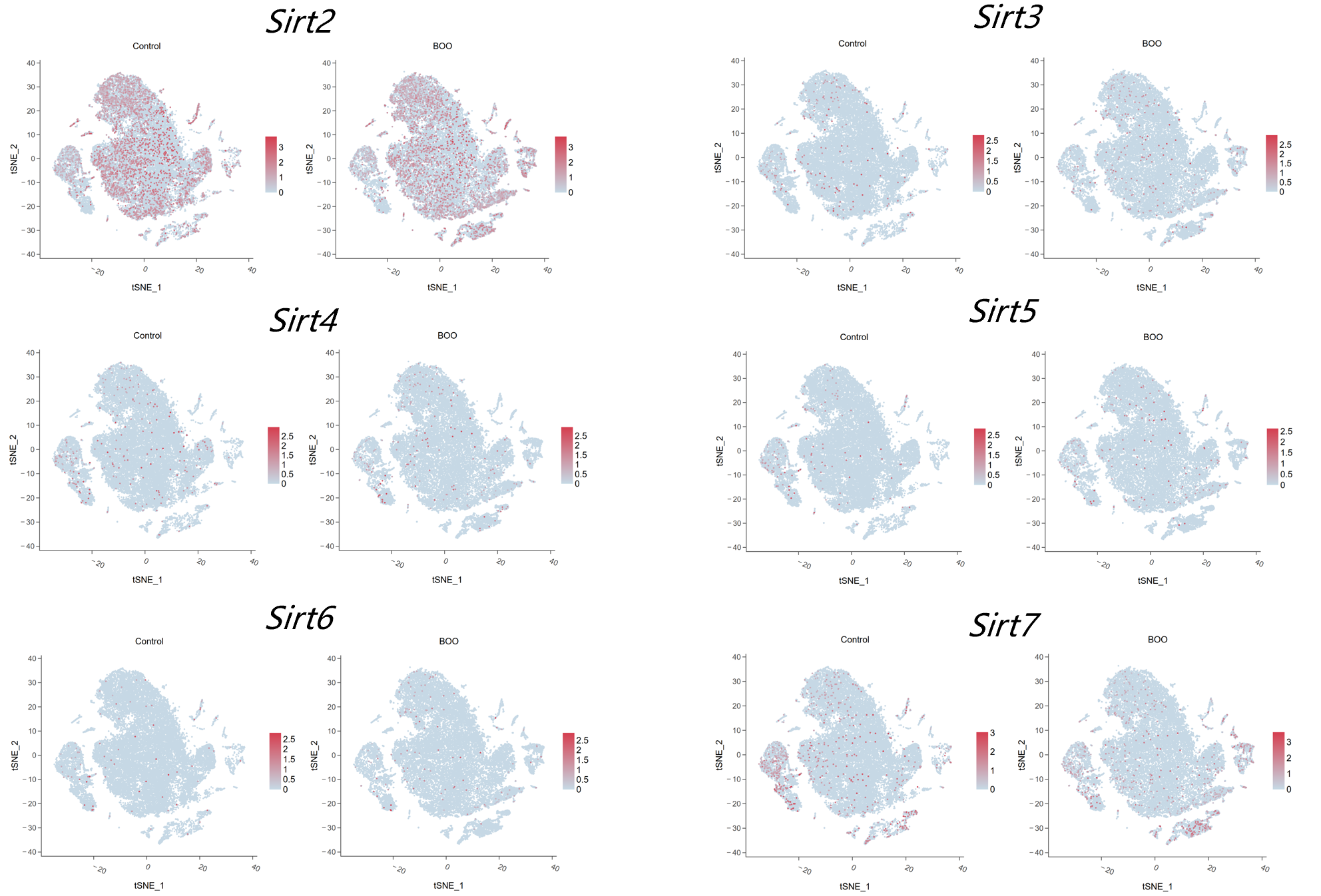


**Figure S1.** Gene expression patterns of SIRT family expression between control and fibrotic bladder tissues


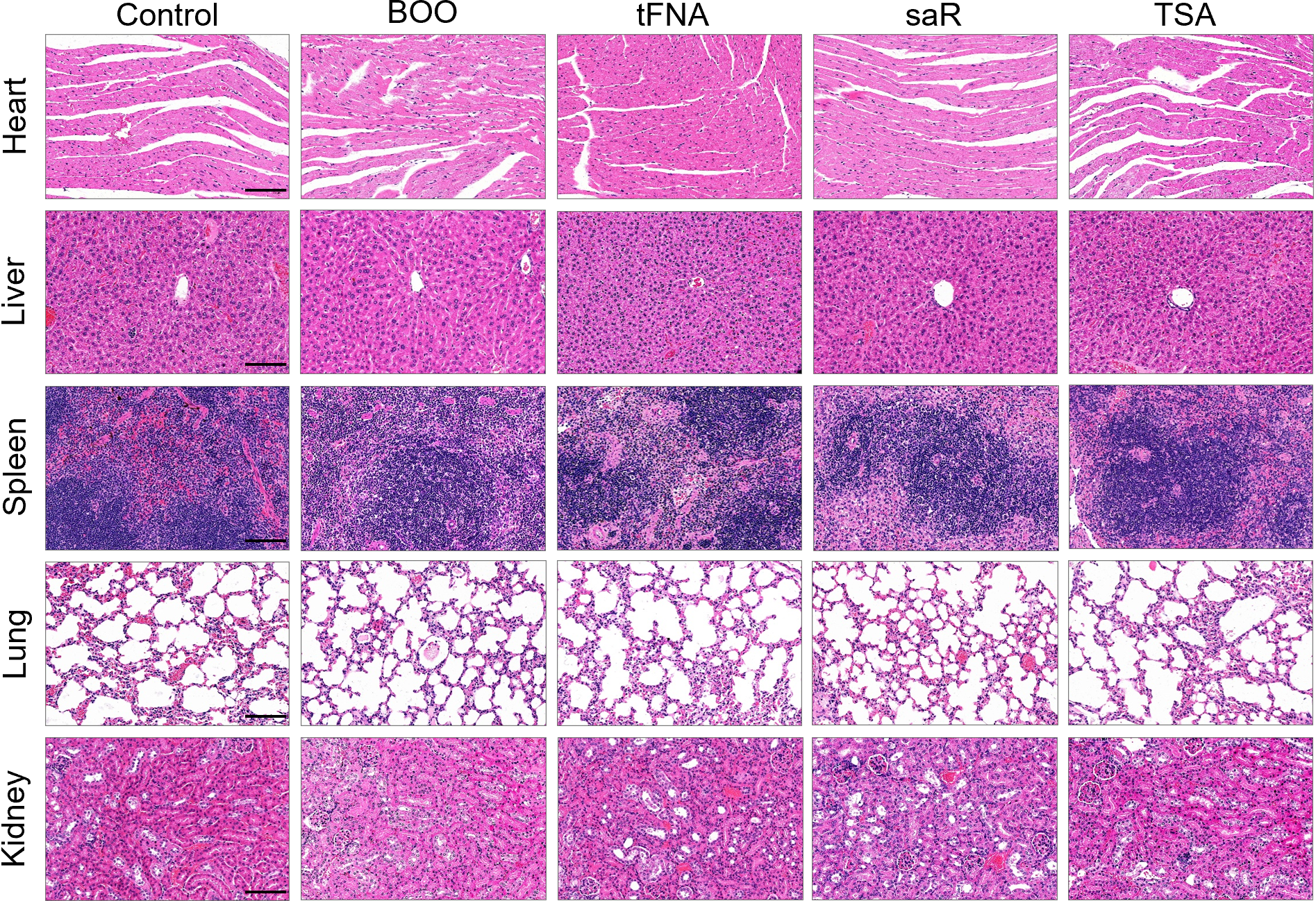


**Figure S2.** H&E staining of heart, lung, liver, spleen, and kidney in five groups to identify the non-toxicity of tFNA, SaR, and TSA to vital organs in vivo (scale bar=100 μm).
